# Viral Communities of Shark Bay Modern Stromatolites

**DOI:** 10.3389/fmicb.2018.01223

**Published:** 2018-06-13

**Authors:** Richard Allen White, Hon L. Wong, Rendy Ruvindy, Brett A. Neilan, Brendan P. Burns

**Affiliations:** ^1^Institute of Biological Chemistry, Washington State University, Pullman, WA, United States; ^2^Crop and Soil Sciences, Washington State University, Pullman, WA, United States; ^3^Plant Pathology, Washington State University, Pullman, WA, United States; ^4^Australian Centre for Astrobiology, University of New South Wales, Sydney, NSW, Australia; ^5^RAW Molecular Systems (RMS) LLC, Spokane, WA, United States; ^6^School of Biotechnology and Biomolecular Science, University of New South Wales, Sydney, NSW, Australia

**Keywords:** ssDNA viruses, viral defense, CRISPR-Cas, BREX, Shark Bay, stromatolites, viral metagenomics

## Abstract

Single stranded DNA viruses have been previously shown to populate the oceans on a global scale, and are endemic in microbialites of both marine and freshwater systems. We undertook for the first time direct viral metagenomic shotgun sequencing to explore the diversity of viruses in the modern stromatolites of Shark Bay Australia. The data indicate that Shark Bay marine stromatolites have similar diversity of ssDNA viruses to that of Highbourne Cay, Bahamas. ssDNA viruses in cluster uniquely in Shark Bay and Highbourne Cay, potentially due to enrichment by phi29-mediated amplification bias. Further, pyrosequencing data was assembled from the Shark Bay systems into two putative viral genomes that are related to *Genomoviridae* family of ssDNA viruses. In addition, the cellular fraction was shown to be enriched for antiviral defense genes including CRISPR-Cas, BREX (bacteriophage exclusion), and DISARM (defense island system associated with restriction-modification), a potentially novel finding for these systems. This is the first evidence for viruses in the Shark Bay stromatolites, and these viruses may play key roles in modulating microbial diversity as well as potentially impacting ecosystem function through infection and the recycling of key nutrients.

## Introduction

Viruses represent the largest genetic repository and most abundant host-associated replicating entities on the planet (Breitbart and Rohwer, 2005; Suttle, 2005, 2007). Viruses infect all living organisms and viruses have been proposed to influence critical biochemical processes, such as photosynthesis and carbon fixation (Suttle, 2005, 2007; Thompson et al., 2011). Environmental viral metagenomics (i.e., viromics) has revealed that >90% of genes are hypothetical or uncharacterized (Angly et al., 2006), and thus it is likely that new genes will be found amongst viruses. A recent metagenomic study that exhaustively analyzed 3,042 geographically diverse samples revealed extensive global viral diversity, including recovering ∼125,000 partial DNA viral genomes, and yet more than 75% of the viral genes were hypothetical or uncharacterized (Paez-Espino et al., 2016). This indicated that more than two-thirds of all viral protein coding genes have currently no known function. The field of viromics currently has many tools for obtaining genomes and benchmarking (Roux et al., 2017), quantification of dsDNA and ssDNA viruses (Roux et al., 2016), and is truly coming of age (Sullivan et al., 2017). Viruses play a key role in carbon cycling representing >20% of all microbial biomass lysed daily in marine ecosystems (Suttle, 2007). This massive genetic repository of billions of uncharacterized and hypothetical genes is formidable, however, linking viruses to ecosystems could provide greater understanding of the role of viruses in global processes.

Previous viral metagenomic studies have suggested that linking various viral genotypes to certain environments to establish viral biogeography is challenging. Often the same viral genotype is found in a variety of ecosystems suggesting that viruses have a cosmopolitan distribution (Breitbart and Rohwer, 2005). A viral metagenomic study that contrasted this concept of cosmopolitan viral biogeography suggested that viral ecotypes do exist in nature (Desnues et al., 2008). It was found that single-stranded DNA microphages from Highbourne Cay stromatolites were endemic and these specific viruses were not found among any other cross-examined ecosystem, including marine, freshwater, terrestrial or metazoan-associated systems. However, this is the only study to date that exists for viral communities among modern microbialites (Desnues et al., 2008).

However, the use of multiple-displacement amplification (MDA) using phi29 polymerase has been well documented to bias amplification toward ssDNA viruses over dsDNA viruses (Kim and Bae, 2011). This can make viral biogeography analyses challenging as absolute quantification of viral ecotypes can be difficult due to this amplification bias, however, general diversity is still maintained (Kim and Bae, 2011). Due to strand displacement events, the phi29 polymerase appears to amplify circular DNA more efficiently than linear DNA in diverse nucleic acid pools found in viromes in environmental ecosystems (Kim and Bae, 2011). A recent study found on average that phi29 polymerase amplification bias toward ssDNA viruses was systematically over-represented >10-fold, and that this method on average captured 2–15 times more ssDNA viral genomes (Roux et al., 2016). However, many studies have used this method (phi29 mediated amplification) to selectively enrich and amplify ssDNA viruses and measure diversity in ocean water (Rosario et al., 2009a), reclaimed water (Rosario et al., 2009b), human feces (Reyes et al., 2010), and modern microbialites (Desnues et al., 2008).

Modern stromatolites are analogs to early microbial ecosystems, some dating back 3.5 billion years (Gya) (Dupraz and Visscher, 2005; Van Kranendonk et al., 2008; Dupraz et al., 2009). However, modern marine stromatolites are less extensively distributed compared to the early Earth (Van Kranendonk et al., 2008), with Shark Bay and Highbourne Cay harboring some of the most well-studied examples. Several studies have characterized the extensive microbial diversity in the Shark Bay ecosystem, including novel bacterial, archaeal, and eukaryotic groups (Burns et al., 2004; Goh et al., 2009; Edgecomb et al., 2014; Wong et al., 2015, 2017; Ruvindy et al., 2016; Suosaari et al., 2016). To date no study has delineated the viral contribution to overall biological diversity of the modern stromatolites of Shark Bay, and the aim here was thus to describe the diversity of viruses in Shark Bay stromatolites for the first time. This was achieved by analyzing the purified viral fraction (e.g., free viral particles) and cellular fraction (i.e., lysogenic/prophage or viruses in active infection amongst the cellular fraction) via filtration then direct shotgun sequencing and comparing to similar microbialite viromes prepared in the same manner (e.g., Highbourne Cay, Pozas Azules II and Rios Mesquites).

## Materials and Methods

### Sampling, Viral Metagenomic Library Construction, and Sequencing

Columnar stromatolites were collected and sampled in 2009 from the south-eastern shore of Hamelin Pool, Shark Bay, Western Australia (26°25 S, 114°130 E) as described previously (Burns et al., 2004; Ruvindy et al., 2016). Samples were collected at low tide using a sterile spatula. At the time of sampling, the temperature was recorded as 27.4°C, salinity 68 (Practical Salinity Unit; PSU) and pH 7.9. Samples were placed in sterile specimen containers and stored at 4°C during transportation for ∼30 min. DNA was extracted immediately upon sample return. Viral and cellular fraction metagenomes were purified, amplified with MDA via phi29 polymerase, and sequenced as described (Desnues et al., 2008). Briefly, ∼5 g of Shark Bay stromatolite material was shaken in 30 ml of SM buffer (0.1 M NaCl, 1 mM MgSO_4_, 0.2 M Tris pH 7.5, 0.01% gelatin within 0.02 μm filtered seawater for 1 h (Desnues et al., 2008). Filtration was used to separate the microbial fraction from the viral fraction using 0.22 μm filters. The Shark Bay cellular fraction (i.e., microbial cellular fraction) was the stromatolite and cellular material collected on the 0.22 μm filter, and the flow through was considered the viral particle fraction. The viral particle fraction was then further purified using cesium chloride density gradient centrifugation (Thurber et al., 2009), and checked for bacterial and eukaryotic cells using SYBR straining and epifluorescence microscopy (Thurber et al., 2009). Both the viral and microbial fraction DNA were isolated using formamide/CTAB extraction (Sambrook et al., 1989), then amplified with phi29-based MDA via GenomiPhi (GE Healthcare) following the manufacturer’s recommendations. Subsequently, ∼10 μg DNA was sequenced using 454 pyrosequencing (Margulies et al., 2005).

### Quality Control of Sequencing Data and Assembly

The 454-pyrosequencing data (raw SFF files) were converted to FASTQ format and binned by molecular barcode (multiplex identifier). Data were examined for quality using FastQC^1^. Shark Bay metagenome barcodes were removed by Tagcleaner (Schmieder et al., 2010), sequences were trimmed for low quality (>Q_25_), poly-A/T/N tails, de-duplicated (100% extract match), and ambiguous bases/sequences (>100 bp) and sequences with complexity (>70) on entropy scales removed by PRINSEQ (Schmieder and Edwards, 2011). High quality reads for the Shark Bay Virome (not cellular fraction) were assembled in order to find putative viral genomes and increase contig size using Ray DeNovo Assembler using (Kmer size = 31) (Boisvert et al., 2010, 2012, **Table 1**).

**Table 1:** Metagenomic statistics including read analysis, assembly stats, and annotations for MetaVir2 and MG-RAST.

Reads	Viral fraction	Cellular fraction	Assembly	Viral fraction
**Raw data**			**Contigs >100 bp**	
Number	92298	73371	Number	504
Total length (bp)	39623558	31023655	Total length (bp)	149063
Average (bp)	429	423	Average (bp)	295
GC%	48%	44%	N50 (bp)	353
			Median (bp)	172
**After QC data**			Largest (bp)	4099
Number	62294	59805	**Contigs >500 bp**	
Artificial duplicate reads	23699	8842	Number	49
Total length (bp)	28413896	26636206	Total length (bp)	57537
Average (bp)	456	445	Average (bp)	1174
GC%	47%	44%	N50 (bp)	1473
			Median (bp)	924
**MG-RAST predictions**			Largest (bp)	4099
Predicted protein features	39127	50281		
Predicted rRNA features	3321	3746		**Cellular fraction**
Identified protein features	2452	23704	**Contigs >100 bp**	
Identified rRNA features	0	64	Number	N/A
Identified functional categories	2033	21025	Total length (bp)	N/A
Failed QC (duplicates/length)	30,004 (32.51%)	13,566 (18.49%)	Average (bp)	N/A
Unknown	1,000 (1.08%)	197 (0.27%)	N50 (bp)	N/A
Predicted feature	61,294 (66.41%)	59,608 (81.24%)	Median (bp)	N/A
Unknown protein	30,514 (49.78%)	20,191 (33.87%)	Largest (bp)	N/A
Annotated protein	30,780 (50.22%)	38,473 (64.54%)	**Contigs >500 bp**	
Ribosomal RNA	0 (0.00%)	944 (1.58%)	Number	N/A
			Total length (bp)	N/A
**MetaVir2 predictions^∗^**			Average (bp)	N/A
50 on score	6.55%	10.62%	N50 (bp)	N/A
*E*-value (10^-3^) + GAAS	9.04%	15.50%	Median (bp)	N/A
*E*-value (10^-5^) + GAAS	7.42%	12.57%	Largest (bp)	N/A
*E*-value (10^-7^) + GAAS	6.53%	10.54%		


*^∗^Represents a significant viral hit. GAAS is genome length normalization.*

### Annotation and Analysis

High quality reads and viral-assembled contigs were loaded onto MetaVir^2^ and updated using MetaVir2 to analyze the Shark Bay Virome and Shark Bay Cellular fraction (Roux et al., 2011, 2014). Basic local alignment search tool (BLAST) based comparison in MetaVir was implemented (*e*-value ≤ 10^-3^, 10^-5^, 10^-7^) against the NCBI refseq database (updated refseq 2017-01-11), and normalized to genome length using the built-in Genome-relative Abundance and Average Size (GAAS) normalization tool (Angly et al., 2006; Roux et al., 2011). KO EC numbers (directly KEGG mapped), refseq and SEED subsystem annotations, were analyzed by MG-RAST. MG-RAST was used for the main taxonomic and functional annotation (Meyer et al., 2008) of both microbial and viral fractions, and MetaVir2 employed for virome analysis. To search for antiviral gene homologs amongst the viral and cellular fraction, high quality reads were translated to predicted proteins using prodigal (Hyatt et al., 2010), and were annotated against the PFAM/TIGRFAM and KEGG using BLAST, InterProScan 5, and GhostKoala (Jones et al., 2014; Kanehisa et al., 2016).

Principal coordinate analyse (PCA) analysis was undertaken using GAAS outputs from MetaVir2 and R libraries Ecodist (dissimilarity-based functions for ecological analysis), pvclust (hierarchical clustering with *P*-values via Multiscale Bootstrap Resampling), ward clustering, and Bray–Curtis distance metrics at a 1000 replicates against the viromes and microbial fractions for Highboune Cay, Pozas Azules II, Ríos Mesquites microbialites (Desnues et al., 2008).

### Phylogenetic Analysis

Marker gene identification was completed using reference trees provided by MetaVir2 for major capsid protein for *Microviridae* (VP1), auxiliary metabolic gene (AMG) phoH which is widespread in phage genomes but whose function remains unknown (Goldsmith et al., 2011), and replication-associated protein (Rep) found in ssDNA viruses.

Of the VP1 contigs, those with sequence length lower than 160 were deleted. Reference viral replication proteins (phoH, VP1, Rep) and proteins obtained from the Shark Bay virome (viral fraction) were aligned using MUSCLE (Edgar, 2004), and alignment gaps were removed with UGENE^3^ (Okonechnikov et al., 2012). Maximum likelihood phylogenetic trees were constructed using IQ-TREE v. 1.6.1 with a total of 1000 bootstrap replicates, and visualized with iTOL (Letunic and Bork, 2016; Hoang et al., 2017).

### Data Availability

The assembled data Shark Bay virome and microbial fraction have been deposited in MetaVir and are available under project names “Shark Bay Virome” and “Shark Bay Microbes,” and additionally in MG-RAST as “Shark Bay Virome,” and “Shark Bay Microbes.” All codes and scripts can be found on github.com/raw937. Both pre-assembled and assembled reads have been deposited in the Sequence Read Archive (SRA) under accession numbers SRR7160500 and SRZ187061, and BioProject identifier “Viral communities of Shark Bay modern stromatolites” (PRJNA471212).

## Results and Discussion

### General Properties of the Shark Bay Stromatolite Cellular and Viral Fraction Metagenomes

DNA sequences for viral and cellular fractions from Shark Bay stromatolites were determined for viral homology and taxonomy using MetaVir2, and MG-RAST for functional annotation. Both the cellular and viral fraction have >50,000 sequences of ∼400 bp, with between 26 and 28 Mbp total sequence length (**Table 1**). The viral fraction contained 50% annotated proteins with another 50% unknown proteins with no rRNA sequences, whereas the microbial fraction contained 64% annotated proteins with 33% unknown and 1.5% rRNAs based on MG-RAST (**Table 1**). The lack of rRNA in the viral fraction, negative PCR results for bacterial 16S rDNA, and epifluorescence microscopy indicating no cells after filtration and CsCl gradient, suggests a relatively pure viral fraction. MetaVir2 predicted viral sequences based on BLAST to refseq (2017-01-11) found that regardless of the *e*-value (10^-3^, 10^-5^, 10^-7^ with GAAS normalization) that >5% have a significant viral hit to known viruses within the database, whereas the cellular fraction had >10% for significant viral hits (**Table 1**). An *e*-value of 10^-5^ with GAAS normalization was chosen for all further taxonomic and viral genome size estimation using MetaVir2, and an *e*-value of 10^-5^ for MG-RAST functional annotation for its conservative value while providing the most significant hits to known databases. Assembly was completed on the viral fraction only in an attempt to find circular ssDNA putative genomes and longer contigs (**Table 1**). Ray assembly of the Shark Bay viral fraction yielded few contigs (49 at >500 bp, 500 at >100 bp) suggesting sparse sampling of the available ssDNA viruses found at Shark Bay (**Table 1**).

### Shark Bay Stromatolite Virome and ssDNA Virus Diversity

**FIGURE 1:**
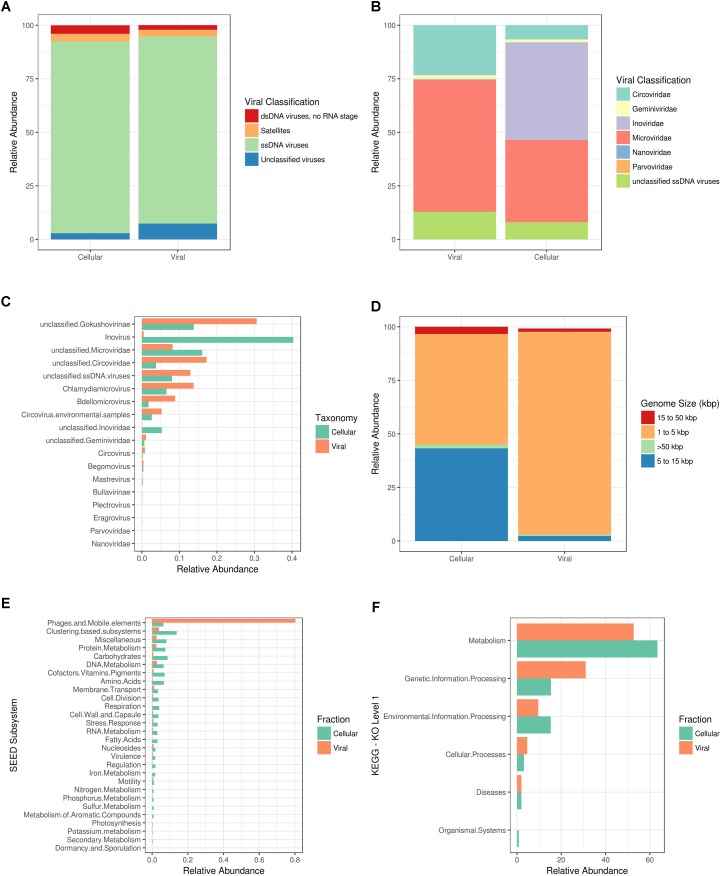
Shark Bay viral and cellular fraction comparison. MG-RAST Functional annotations using KEGG (KO) and SEED where based on BLAT based comparison (*e*-value ≤ 10^-5^) against respective database. **(A)** Viral taxonomic classification characterization by nucleic acid state in MetaVir2. **(B)** Viral taxonomic classification characterization by viral family in MetaVir2. **(C)** Viral taxonomic classification characterization by viral genus in MetaVir2 by cellular or viral fraction metagenome. **(D)** GAAS predictive viral genome size estimations in MetaVir2. **(E)** SEED subsystem functional annotations in MG-RAST. **(F)** KEGG (KO) level 1 functional annotations in MG-RAST. MetaVir2 viral (BLAST) based comparison (*e*-value ≤ 10^-5^) against NCBI viral refseq database and normalized to genome length using the built-in Genome-relative Abundance and Average Size (GAAS) normalization tool (Angly et al., 2006).

ssDNA viruses were the most abundant amongst the viral sequences due to the enrichment of phi29-based MDA of the Shark Bay stromatolite viral fraction (i.e., free viral particles). It is important to note that few sequences within the Shark Bay virome had representative annotated hits to known viral genomes, and downstream analyses described are based on known annotated viral genomes present within MetaVir2. More than 85% of reads relating to viruses in the viral and cellular fraction from Shark Bay were ssDNA viruses (*e*-value ≤ 10^-5^ with GAAS normalization) (**Figure 1A**). *Microviridae* represented >50% of annotated ssDNA virus sequences within the viral fraction and >30% in the cellular fraction (**Figure 1B**). The cellular fraction had ∼40% *Inoviridae* sequences whereas the viral fraction had <1% (**Figure 1B**). The *Inoviridae* sequences were ∼35% inovirus in the cellular fraction with some unclassified members (**Figure 1C**). *Microviridae* in Shark Bay are likely directly infecting hosts, as *Microvirida*e are rarely lysogenic with the exception of the proviruses that infect *Bacteroidetes* (Krupovic and Forterre, 2011). *Microviridae* as a group are more often found to be lytic than lysogenic, and thus the higher presence of *Microviridae* annotated sequences in the cellular fraction in the present study could potentially be active infection (Szekely and Breitbart, 2016). Chlamydiamicroviruses composed 12% of the *Microviridae* sequences amongst the Shark Bay virome (**Figure 1C**), and these were dominated by subfamily Gokushovirinae at 27% (**Figure 1C**). The gokushoviruses have been shown to be widespread in many marine ecosystems (Hopkins et al., 2014). Amongst the Chlamydiamicroviruses sequences within the Shark Bay virome, some sequences were most similar to chlamydia phage 3 and 4-like sequences (Supplementary Table 1). Bdellomicrovirus sequences also comprised ∼8% of the Shark Bay *Microviridae* sequences, and this virus is known to infect the bacterium *Bdellovibrio*.

Genome-relative abundance and average size normalization allowed for general prediction of the relative viral genome size for both cellular and viral fraction using MetaVir2 with large differences between the two fractions. Greater than 90% of the predicted genome sizes within the viral fraction were 1–5 kbp (**Figure 1D**). In contrast ∼50% was represented in the predictive genome size of 1–5 kbp (**Figure 1D**) in the cellular fraction. The other ∼40% were predicted to be viral genomes of 5–15 kbp (**Figure 1D**). The likely reason for this selectively lower viral genome is due to the phi29-mediated amplification, and future work is needed to amplify the majority of viral nucleic acids without dsDNA or ssDNA biases.

The top functional genes present and SEED subsystems for Shark Bay viral fraction included >80% in related to phages, prophages, transposable elements, and/or plasmids (**Figure 1E**). KEGG KO level 1 annotation for both viral and cellular fraction suggested metabolism >40% represented most functional calls by KEGG (**Figure 1F**). KEGG EC included phage viral structural genes (e.g., capsids, tails, **Table 2**) and DNA metabolism related genes (e.g., DNA 5-cytosine methylase, ribonucleoside-diphosphate reductase, and DNA helicase, **Table 2**). These functional genes potentially facilitate key processes in the Shark Bay stromatolite host communities, including viral attachment, protection of viral genetic material, and viral replication (Hofer, 2016).

**Table 2:** KEGG ontology (KO) EC numbers for the Shark Bay virome annotations reads.

EC description	EC number	Abundance
DNA (cytosine-5-)-methyltransferase	2.1.1.37	36
Ribonucleoside-diphosphate reductase	1.17.4.1	11
DNA helicase	3.6.4.12	8
GDP-mannose 4,6-dehydratase	4.2.1.47	5
Riboflavin kinase	2.7.1.26 2.7.7.2	5
Methyltransferases	2.1.1.-	4
Carbon-oxygen lyases/Hydro-lyases	4.2.1.-	3
Spermidine dehydrogenase	1.5.99.6	3
DNA-directed DNA polymerase	2.7.7.7	2
Nicotinamidase	3.5.1.19 3.5.1.-	2
Ribonucleoside-triphosphate reductase	1.17.4.2	2
Nucleotidyltransferases	2.7.7.-	2
Thymidylate synthase	2.1.1.148	2
Sarcosine oxidase	1.5.3.1	2
Leucyl aminopeptidase	3.4.11.1	2
Xanthine dehydrogenase	1.17.1.4	1
Aspartate carbamoyltransferase	2.1.3.2	1
Deoxyuridine-triphosphatase	3.6.1.23	1
Histidine permease	3.6.3.21	1
Amidophosphoribosyltransferase	2.4.2.14	1
Indolepyruvate decarboxylase	4.1.1.74	1
NAD+ synthase (glutamine-hydrolysing)	6.3.5.1	1
Allantoicase	3.5.3.4	1
Peptidyl-dipeptidase A	3.4.15.1	1
Dihydrofolate reductase	1.5.1.3 2.1.1.45	1
GDP-L-fucose synthase	1.1.1.271	1
DNA-directed RNA polymerase	2.7.7.6	1
UDP-*N*-acetylglucosamine-1-carboxyvinyltransferase	2.5.1.7	1
Tryptophan synthase	4.2.1.20	1
3-oxoacyl-[acyl-carrier-protein] reductase	1.1.1.100	1
Oxidoreductases with NAD+ or NADP+ as acceptors	1.1.1.-	1
Protoporphyrinogen oxidase	1.3.3.4	1


*MG-RAST cut-offs were at 1e-5 *e*-value, min% identity of 60%, and minimum alignment length of 50 bp.*

Surprisingly, no haloarchaeal viruses were identified here, although haloarchaea are prominent in the Shark Bay microbialite systems (Burns et al., 2004; Allen et al., 2009; Wong et al., 2017), and have been hypothesized to ‘fill the niche’ as potentially major players in nutrient cycles. Despite the lack of haloarchaeal viruses/phage amongst our data, there were unclassified sequences from assembled contigs ∼25% or ∼1.08% unassembled reads (**Table 1**) with no hits to public databases, and haloarchaeal viral genes could putatively be amongst these. In addition, two of the putative viral contigs which contain only replication protein genes could be associated with haloarchaea.

### Comparison Between Shark Bay Virome and Microbial Fraction With Other Microbialite Ecosystems

**FIGURE 2:**
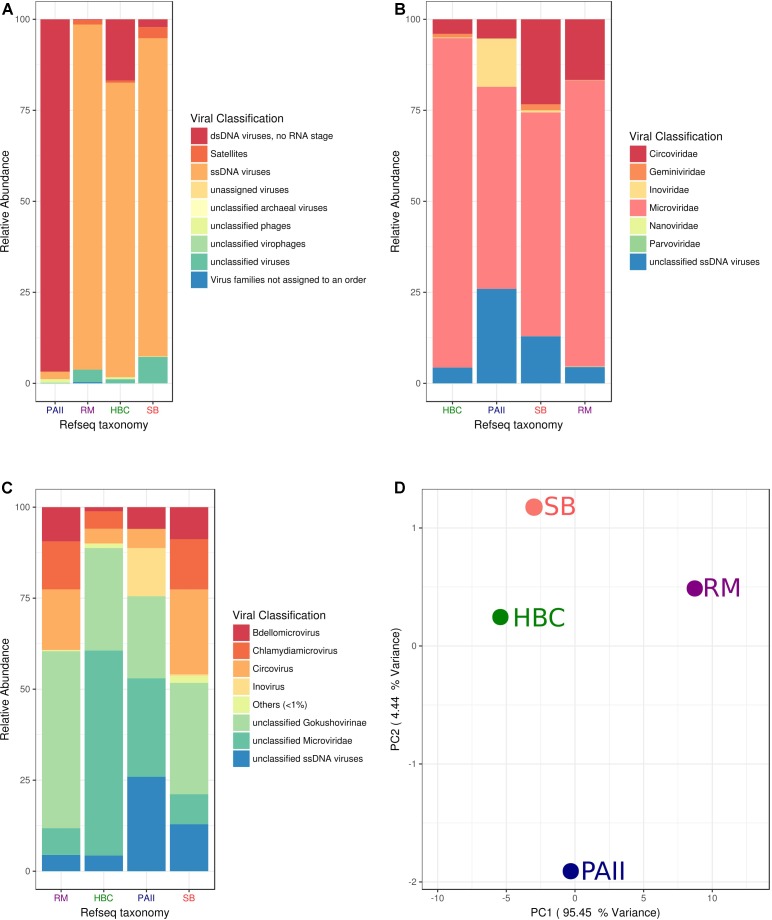
Viral fraction comparison Shark Bay against Highbourne Cay, Rios Mesquites and Pozas Azules II microbialite sites. **(A)** Viral taxonomic classification characterization by nucleic acid state in MetaVir2. **(B)** Viral taxonomic classification characterization by viral family in MetaVir2. **(C)** Viral taxonomic classification characterization by viral genus in MetaVir2. **(D)** Principal coordinate analyses (PCA) comparing the viral diversity in disparate stromatolite locations. PCA were constructed from similarity matrices utilizing protein coding sequence recruitment using NCBI viral refseq database (refseq update 2017-1-11) and normalized to genome length using the built-in GAAS. Proportion variance (PC) was explained by each component printed next to the PC1/PC2 axes labels. MetaVir2 viral (BLAST) based comparison (*e*-value ≤ 10^-5^) against NCBI viral refseq database and normalized to genome length using the built-in Genome-relative Abundance and Average Size (GAAS) normalization tool (Angly et al., 2006). MG-RAST Functional annotations using KEGG (KO) and SEED where based on BLAT based comparison (*e*-value ≤ 10^-5^) against respective database. SB, Shark Bay; HBC, Highbourne Cay; PAII, Pozas Azule II; RM, Rio Mesquites.

Shark Bay viral and cellular fraction metagenomes were compared to previously reported microbialite ecosystems (Highbourne Cay, Pozas Azules II, and Rios Mesquites). *Microviridae* sequence dominance within the Highbourne Cay viromes has been previously noted (Desnues et al., 2008), and the Shark Bay viromes were very similar in viral taxonomic composition (**Figure 2A**). Highbourne Cay, Rios Mesquites and Shark Bay viromes had >80% of sequences as ssDNA viruses (**Figure 2A**), which may be attributed to phi29-mediated bias. Of those ssDNA sequences, Highbourne Cay, Rios Mesquites and Shark Bay >50% were of *Microviridae-*like sequences, with Shark Bay virome having >20% of circoviridae origin (**Figure 2B**). Pozas Azules II had >90% as dsDNA virus sequences (Desnues et al., 2008). Data here was normalized to 100% for ssDNA virus sequences for Pozas Azules II to compare ssDNA viruses across all four sites, with Pozas Azules II possessing >50% *Microviridae-*like sequences after normalization comprising the bulk of the ssDNA sequences in that ecosystem. There were some compositional differences between Shark Bay and Highbourne Cay viromes, as Shark Bay had >25% of the sequences with similarity to *Chlamydia* phage 3 and 4 (represented as Chlamydiamicrovirus), whereas Highbourne Cay had >20% of the sequences with similarity to unclassified *Microviridae* (**Figure 2C**). Shark Bay ssDNA virus sequences were highly similar and clustered with Highbourne Cay viromes (**Figure 2D**). The presence of the high levels of ssDNA sequences explained the majority of principal coordinate clustering observed (**Figure 2D**).

### Long Viral Contigs and Putative Viral Genomes in Shark Bay Stromatolites

Two of the contigs obtained from the Shark Bay viral fraction (the largest contigs) have no BLAST hits to any reference sequence in NCBI databases. SB contig-2000010_3827 (3827 bp) and contig-4_4099 (4099 bp) have low *E*-values (∼1 × 10^3^) to hypothetical proteins or recombinases (**Table 3**). Two of the contigs (7000012_1784 and 1000003_2028) are putative viral genomes as they are circular and have homologs to replication protein (e.g., *Rep*), and related to Sewage-associated circular DNA virus-30 and Sewage-associated circular DNA virus-18 respectively (**Table 3**). Both of these viruses, Sewage-associated circular DNA virus-30 and Sewage-associated circular DNA virus-18, are novel circular replication-associated protein encoding single-stranded (CRESS) DNA viral genomes (Kraberger et al., 2015). These viruses are classified now as novel *Genomoviridae* within CRESS family (Krupovic et al., 2016), whereas the hosts of these viruses are unknown most members infect eukaryotes associated with infecting plants and animals not bacteria. These viruses could be introduced to microbialite systems by seabirds endemic to an area (Desnues et al., 2008), a scenario that could also potentially be occurring in Shark Bay.

**Table 3:** BLAST results against NCBI against long contigs and putative viral genomes in the Shark Bay viral fraction.

	Gene	Max score	Total score	Query cover %	*E*-value	Ident %	Accession
**contig-7000012_1784**							
Uncultured virus	Viral replication-associated protein (Rep)	172	172	56.00	1.00E-44	34.00	AUM61732.1
Uncultured virus	Viral replication-associated protein (Rep)	171	171	57.00	4.00E-43	33.00	AUM62051.1
Sewage-associated circular DNA virus-30	Viral replication-associated protein (Rep)	166	166	52.00	2.00E-41	32.00	YP_009117070.1
**contig-1000003_2028**							
Sewage-associated circular DNA virus-18	Viral replication-associated protein (Rep)	170	170	50.00	2.00E-43	31.00	YP_009116898.1
Uncultured virus	Viral replication-associated protein (Rep)	169	169	47.00	1.00E-42	33.00	AUM61781.1
Uncultured virus	Viral replication-associated protein (Rep)	164	164	49.00	2.00E-41	34.00	AUM61982.1
**Contig-1000009_1678**							
Ralstonia picketti	Hypothetical protein	636	636	55.00	0	99.00	WP_024972784.1
Cellulophaga phage phi47:1	Hypothetical protein CDPG_00080	629	629	54.00	0	99.00	AGF91683.1
Cellulophaga phage phi47:1	Hypothetical protein CDPG_00081	276	276	27.00	3.00E-87	100.00	AGF91684.1
contig-1000007_1202							
Pseudanabaena sp. ’Roaring Creek	Hypothetical protein	45.8	45.8	13.00	0.019	45.00	WP_055077263.1
Synechococcus sp. PCC 7502	Hypothetical protein	42	42	11.00	0.093	50.00	WP_015169903.1
Oscillatoriales cyanobacterium USR001	Hypothetical protein BCD67_24715	39.3	39.3	15.00	2.1	31.00	OCQ97517.1
Nocardia transvalensis	Patatin	41.2	41.2	22.00	3.7	34.00	WP_040746262.1
**contig-4_4099**							
Uncultured prokaryote	Hypothetical protein	48.1	48.1	11.00	0.037	27.00	CRY97485.1
Uncultured prokaryote	Hypothetical protein	41.6	41.6	3.00	5.3	46.00	CRY96835.1
*Actinoplanes subtropicus*	Recombinase family protein	42.7	42.7	4.00	6.7	39.00	WP_084599775.1
contig-2000010_3827							
Uncultured prokaryote	Hypothetical protein	75.9	75.9	11.00	6.00E-11	34.00	CRY96346.1
*Azospirillum sp. 51_20*	Hypothetical protein	72.4	72.4	4.00	2.00E-09	57.00	OLA80278.1
*Sphingopyxis terrae*	Hypothetical protein	69.7	69.7	4.00	7.00E-09	52.00	WP_082813420.1
**contig-1000002_2208**							
*Tateyamaria omphalii*	DNA ligase-associated DEXH box helicase	42.4	42.4	18.00	4.5	29.00	WP_076628122.1
*Streptomyces*	MULTISPECIES: serine/threonine protein kinase	42	42	19.00	7.1	28.00	WP_103536509.1
Contig-9_1603							
No hits							
Contig-8_1554							
No hits							
Contig-7_1473							
No hits							


### Environmental Relevance of ssDNA Viruses in Shark Bay

Eukaryotic grazers represent a potential destabilizing factor by grazing nutrient rich stromatolites, including Shark Bay stromatolites (Farmer, 1992; Edgecomb et al., 2014). Marine geminiviruses and circoviruses infect a wide range of eukaryotic organisms, including protists, marine arthropods, and other grazers (Rosario et al., 2009a; Saccardo et al., 2011). We hypothesize that the marine viruses identified in the present study may infect eukaryotic grazers, with the viruses acting act as a top–down control. Viral mediated lysis of eukaryotic grazers may help stabilize the stromatolite ecosystems in Shark Bay, that could otherwise be disrupted by excessive grazing. While the aforementioned viruses are obligate eukaryotic-associated, the Shark Bay virome contains viruses similar to those also capable of infecting bacteria, such as the *Bdellomicroviruses* (**Figure 2**). Future studies are needed in eukaryotic circular Rep-encoding ssDNA (CRESS) viruses within Shark Bay stromatolites and other environments, to measure the rates of viral mediated lysis of eukaryotes amongst phytoplankton and zooplankton, as such top–down control could have global impacts of nutrient cycling in the ocean. Single cell techniques may elucidate such viral-host (e.g., protist-viral) interactions (Gavelis et al., 2015). Recent studies employing iTag deep amplicon sequencing of bacterial communities in Shark Bay microbialites indeed identified *Bdellovibrio* as one of the prominent community members (Wong et al., 2015). Thus, the potential for infection by this group of viruses with known microbialite hosts in Shark Bay is present, and future work will help clarify the extent of this process in these ecosystems.

### Microbial Viral Defense Mechanisms in Shark Bay Stromatolites

**FIGURE 3:**
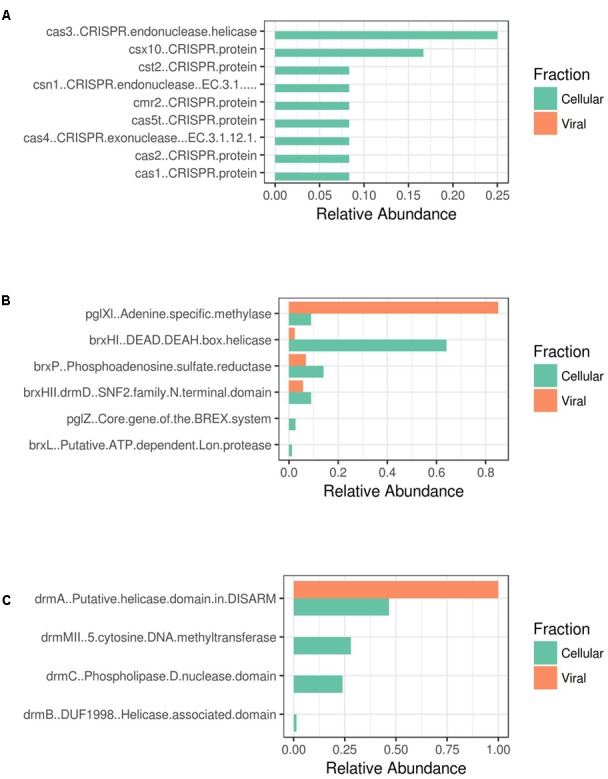
Antiviral resistance genes amongst Shark Bay viral and cellular fraction. **(A)** CRISPR-Cas associated genes **(B)** BREX associated genes **(C)** DISARM associated genes. The relative abundance is the number of hits from GhostKoala and the Pfam database using InterProScan 5.

Furthermore, metagenomic analysis of Shark Bay stromatolites has revealed putative viral defense mechanisms present. Evidence of CRISPR-Cas, BREX (bacteriophage exclusion), and DISARM (defense island system associated with restriction-modification) (Goldfarb et al., 2015; Ofir et al., 2018) in both the viral and cellular fraction metagenomes from Shark Bay were found in the present study (**Figure 3**). The genetic basis of one such mechanism, CRISPR (clustered, regularly interspaced, short palindromic repeat systems), was also identified in the Shark Bay microbial metagenomes (Ruvindy et al., 2016). When compared to the viral fraction, an enrichment of CRISPR-Cas genes was observed in the cellular fraction with none in the viral fraction (**Figure 3A**). BREX genes are also more abundant in the cellular fraction than the viral fraction (**Figure 3B**), with the viral fraction having an abundance of Adenine-specific methylase that may putatively be used against host methylation of viral DNA (**Figure 3B**). DISARM genes were also enriched in the cellular vs. viral fraction (**Figure 3C**), including the primary helicases. This is the first evidence of DISARM and BREX in metagenomes enriched in cellular fractions associated with stromatolites. CRISPR systems have been identified as an adaptive microbial immune system that provides acquired immunity against viruses (Horvath and Barrangou, 2010), and thus there may be an interplay between the viral populations identified in the present study and the defense mechanisms characterized in host Shark Bay populations.

A recent study of freshwater microbialites in Pavilion Lake, BC suggested that T4-like phage (e.g., *Myoviridae*) and large algal viruses (e.g., Phycodnaviridae) dominated the viral sequences in the water compared to microbialites, whereas the microbialites possessed genes related to viral defense (e.g., CRISPR, phage shock and phage excision) (White et al., 2016a). Future metagenomic sequencing efforts in Shark Bay should target in detail the two novel viral defense systems of BREX and DISARM, to help complete our understanding of the viral load in the modern microbialites of Shark Bay.

### Phylogenetics of Shark Bay Stromatolite Viral Fraction

**FIGURE 4:**
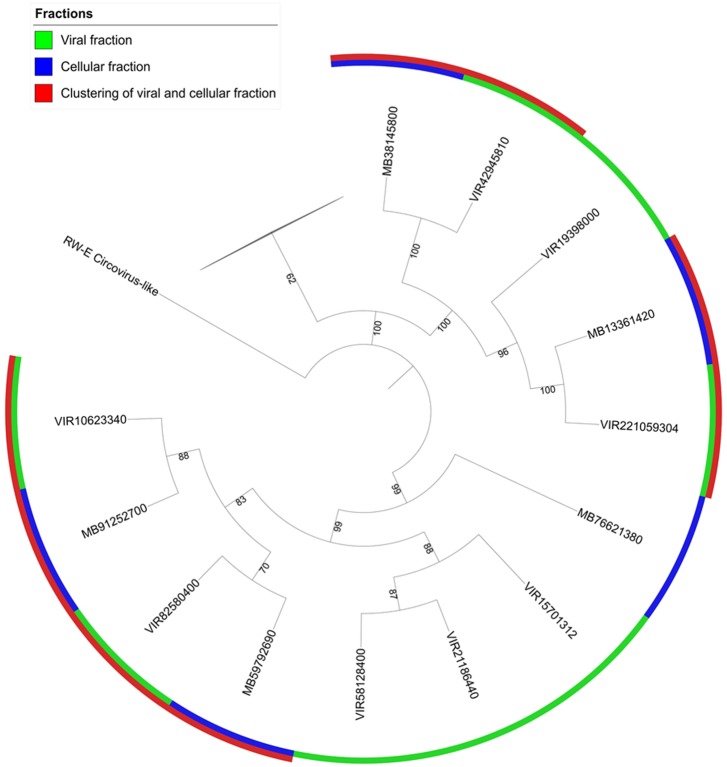
Phylogenetic analysis of microbial and viral fractions from Shark Bay stromatolites for the Replication-associated protein (Rep) in CRESS viruses. Maximum likelihood phylogenetic tree of *Rep* protein sequences found in CRESS viruses in Shark Bay stromatolites. Protein sequence alignments were performed using MUSCLE and gaps in alignment were removed with UGENE. The tree was constructed with IQ-TREE v. 1.6.1 with 1000 bootstrap replicates and was visualized with iTOL. Number indicates bootstrap values, nodes with bootstrap values lower than 70 were not shown and represented by the collapsed branch. The collapsed branches in this figure represent reference sequences from Rosario et al. (2009a) for the replication (rep) protein that have lower than 70 bootstrap values.

Circovirus-like viruses were also detected in Shark Bay viromes (**Figure 2C**), which are thought to associate with and potentially infect eukaryotic grazers, such as insects, snails, and other marine arthropods (Rosario et al., 2009a). Closely related Rep-encoding sequences were found in both the cell and viral fractions, indicative of the potential active infection amongst bacterial cells (**Figure 4**). Phylogenetic tree constructed indicates there are four pairs of contigs from microbial fraction and viral fraction that clustered together (**Figure 4**). These viruses in the viral fraction were found amongst the microbialite itself suggesting potential active infection by ssDNA could be occurring in Shark Bay stromatolites. Eukaryotic grazers can feed on cyanobacterial mats that are the basal unit of stromatolite formation, stability and construction, and thus the presence of viruses that infect and inhibit these grazers could be a top–down control and maintenance of the stromatolite ecosystem in Shark Bay.

**FIGURE 5:**
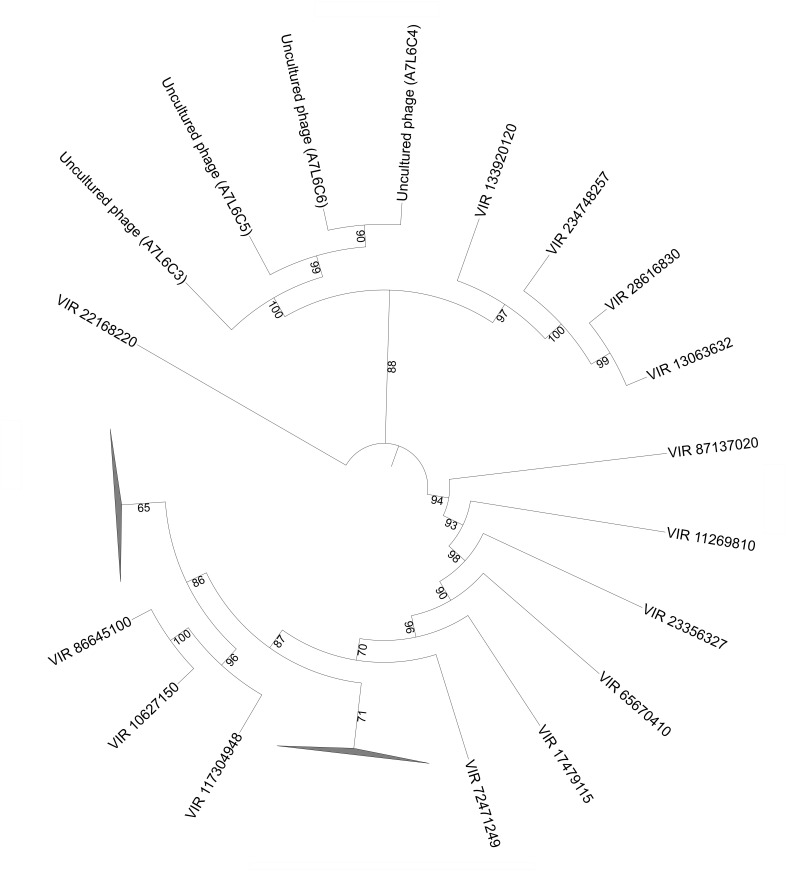
Phylogenetic analysis of viral fractions from Shark Bay stromatolites for the major capsid protein (VP1) in *Microviridae* viruses. Maximum likelihood phylogenetic tree of VP1 protein sequences obtained from *Microviridae* viruses in Shark Bay stromatolites. Reference sequences were retrieved from the Uniprot database. Short sequences (<160 amino acid) were removed prior to alignment. Alignments were performed using MUSCLE and gaps in alignment were removed with UGENE. The tree was constructed with IQ-TREE v. 1.6.1 with 1000 bootstrap replicates and was visualized with iTOL. Number indicates bootstrap values, nodes with bootstrap values lower than 70 were not shown and represented by the collapsed branch. The collapsed branches in this figure represent reference sequences from Desnues et al. (2008) for major capsid protein (VP1) that have lower than 70 bootstrap values.

Viral capsid protein gene (VP1) for microphages (i.e., *Microviridae*) was first described in stromatolites from Highbourne Cay, Rios Mesquites, and Pozas Azules II (Desnues et al., 2008). In the present study, the VP1 sequences obtained in the Shark Bay viral fraction have expanded the quantity of known VP1 sequences (**Figure 5**), and BLAST results also indicate that the branches are derived from uncultured phages. The potential hosts for these VP1-like sequences are likely bacterial hosts over eukaryotes due to their homology to uncultured phages.

**FIGURE 6:**
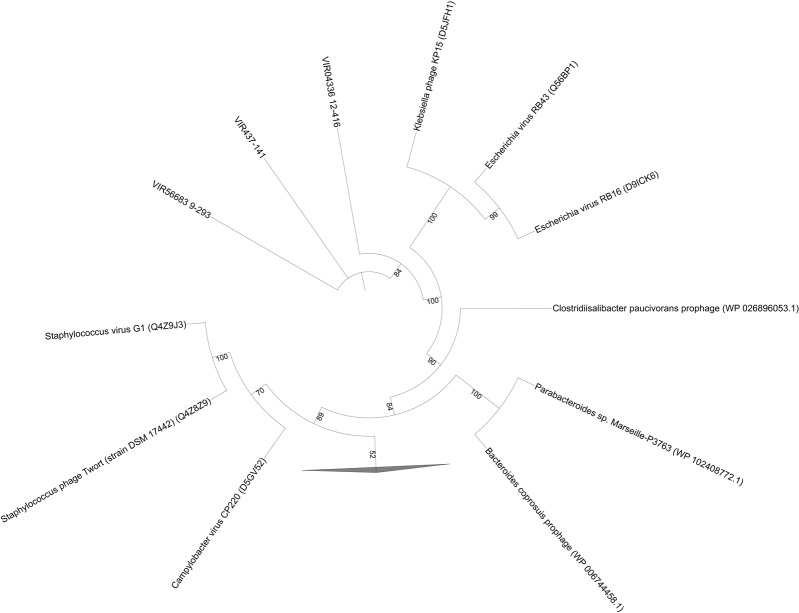
Phylogenetic analysis of viral fractions from Shark Bay stromatolites for phoH (of the pho regulon) in dsDNA viruses. Maximum-likelihood tree of phoH protein sequences obtained from dsDNA viruses. Reference sequences were retrieved from Uniprot database. Alignments were performed using MUSCLE and gaps in alignment were removed with UGENE. The tree was constructed with IQ-TREE v. 1.6.1 with 1000 bootstrap replicates and was visualized with iTOL. Number indicates bootstrap values, nodes with bootstrap values lower than 70 were not shown and represented by the collapsed branch. The collapsed branches in this figure represent reference sequences from Goldsmith et al. (2011) for the phosphate starvation inducible protein (phoH) that have lower than 70 bootstrap values.

As mentioned above, PhoH is a viral AMG with unknown function. Phosphorus limitation has been reported in previous studies in Shark Bay (Smith and Atkinson, 1984; Atkinson, 1987; Wong et al., 2015), as well as in freshwater microbialites (White et al., 2015, 2016a,b). The pho regulon as well as a high abundance of alkaline phosphatases were found amongst the columnar stromatolite (microbial fraction) analyzed here (**Figure 6**), as well as in a previous study (Ruvindy et al., 2016). This regulon has also been identified in soda lake microbialites of Mexico (Valdespino-Castillo et al., 2014). After further BLAST analysis of the phoH sequences in the present study, it was confirmed by manual examination of the reference genomes that they are viral phoH sequences from prophage in these bacterial genomes (**Table 4** and **Figure 6**). Inducible prophages are often found in marine systems that infect large groups of bacteria including marine aerobic anoxygenic phototrophic bacteria (AAPB) (Zheng et al., 2014). These prophage appeared to be integrated into the genomes of *Clostridiisalibacter paucivorans* and *Bacteroides coprosuis* DSM18011 (**Table 4** and **Figure 6**). Other reference genomes in public databases are in draft form and annotations were unclear and unreliable, as the phoH accessions resided in regions of unknown or hypothetical proteins. While phoH has been found to be a core gene in T4-like phages, its function within ecosystems remains to be elucidated (Roux et al., 2015). However, we acknowledge that further investigation is warranted, and a more complete virome is needed for Shark Bay microbialites in order to ascertain whether phoH genes and complete pho regulons regulate phosphate uptake under low-phosphate conditions within Shark Bay.

**Table 4:** BLAST results against NCBI for phoH sequences in the Shark Bay viral fraction.

phoH sequences	Gene name	Species	Max score	Total score	Query cover %	*E*-value	Ident %	Accession
Sequence VIR56683 9-293	PhoH family protein	*Parabacteroides* sp. Marseille-P3763	110	110	96.00	1.00E-26	56.00	WP_102408772.1
	PhoH family protein	*Bacteroides coprosuis*	110	110	96.00	2.00E-26	58.00	WP_006744458.1
	PhoH family protein	*Defluviitoga tunisiensis*	110	110	97.00	2.00E-26	50.00	WP_045087935.1
Sequence VIR437-141	hypothetical protein D478_16524	*Brevibacillus agri* BAB-2500	112	112	98.00	3.00E-30	56.00	ELK40902.1
	PhoH family protein	*Clostridiisalibacter paucivorans*	119	119	98.00	1.00E-29	60.00	WP_026896053.1
	ATPase	*Bacillus boroniphilus* JCM 21738	107	107	98.00	3.00E-28	54.00	GAE45877.1
Sequence VIR04336 12-416	PhoH family protein	*Moorella thermoacetica*	148	148	98.00	3.00E-40	52.00	WP_075517747.1
	PhoH family protein	*Clostridiisalibacter paucivorans*	147	147	100.00	1.00E-39	52.00	WP_026896053.1
	PhoH family protein	*Alteribacillus iranensis*	145	145	99.00	3.00E-39	50.00	WP_091657652.1


### Potential Role of Viruses in Shark Bay

Viruses are also well known as major players in marine nutrient cycling (Suttle, 2007), and they may also play such a role in Shark Bay systems. All viruses including ssDNA, dsDNA, and RNA viruses, are agents of cellular lysis due to infection and death. We hypothesize that the ssDNA viruses found in Shark Bay may be putative drivers of nutrient cycling mediated through eukaryotic and bacterial cell lysis, which releases dissolved nutrients (e.g., C, P, N, S) to be utilized by other microbial community members as has been shown elsewhere (Bratbak et al., 1992; Gobler et al., 1997; Scanlan and Wilson, 1999; Jover et al., 2014). Viruses in Shark Bay are also likely to replenish dissolved organic carbon (DOC) upon cell lysis, thus also playing a role in the carbon cycle (Bratbak et al., 1992). However, further work is needed to delineate the exact role of viruses in biogeochemical cycling in these communities.

## Conclusion

Data from the present study document for the first-time viral diversity amongst Shark Bay stromatolites. Although complete viral diversity remains to be captured due to potential phi29 polymerase MDA bias toward ssDNA viruses, the virome (i.e., viral fraction) revealed significant ssDNA viral diversity. Future work is needed on the viruses in microbialites and stromatolites of Shark Bay, potentially employing a long-read technology such as PacBio, Oxford Nanopore or Illumina Moleculo (White et al., 2016a), which could result in longer contigs, improved assemblies, and novel viral genomes. Microbialites and stromatolites provide modern models to ancient early complex ecosystems, and the data presented here is of significant value to our understanding of some of the first complex microbial ecosystems on Early Earth. Future work employing deeper sequencing and targeting other viruses will help further our understanding of viral diversity in these ecosystems in addition to the ssDNA viruses described here, and determine their exact contribution to functional complexity in Shark Bay.

## Author Contributions

RAW conducted the data analysis, assemblies, and wrote the manuscript sections. HW and RR conducted the data analysis, phylogenetic analysis, and contributed to manuscript sections. BN and BB coordinated and designed the research, and wrote manuscript sections. All the authors read and approved the manuscript.

## Conflict of Interest Statement

The authors declare that the research was conducted in the absence of any commercial or financial relationships that could be construed as a potential conflict of interest.

## Footnotes

1 www.bioinformatics.babraham.ac.uk/projects/fastqc/

2 http://metavir-meb.univ-bpclermont.fr/

3 http://ugene.net/
